# Prescription of potentially addictive medications after a multilevel community intervention in general practice

**DOI:** 10.1080/02813432.2023.2168125

**Published:** 2023-01-20

**Authors:** Muhunthan Navaratnam, Gunnhild Åberge Vie, Thea Brevik, Bjarne Austad, Cato Innerdal, Linn Okkenhaug Getz, Håvard Kjesbu Skjellegrind

**Affiliations:** a Molde Brygge General Practitioner Office, Molde Municipality, Molde, Norway; b Department of Public Health and Nursing, Faculty of Medicine and Health Sciences, Norwegian University of Science and Technology, NTNU, Trondheim, Norway; c Faculty of Health Sciences and Social Care, Molde University College, Molde, Norway; d Clinic of Surgery, Møre and Romsdal Hospital Trust, Molde Hospital, Molde, Norway; e Molde Municipality, Molde, Norway; f General Practice Research Unit, Department of Public Health and Nursing, Faculty of Medicine and Health Sciences, Norwegian University of Science and Technology, NTNU, Trondheim, Norway

**Keywords:** Opioid, benzodiazepine, z-hypnotic, tapering, inappropriate prescribing, polypharmacy, public health

## Abstract

**Objective:**

To evaluate the long-term effects of a multilevel community intervention to improve the quality of prescription practice of potentially addictive medications (PAMs).

**Design:**

We conducted a retrospective study, using anonymized data from the Norwegian prescription registry.

**Setting:**

Based on an initiative from the GPs in Molde Municipality in Norway, a multilevel community intervention was initiated by the municipal chief physician in 2018. The intervention targeted GPs, patients, and the public.

**Subjects:**

We retrieved prescription data from 26 of 36 GPs.

**Main outcome measures:**

By using the standardized defined daily dose (DDD), we compared prescription of three groups of PAMs from before the intervention (2017) throughout the intervention in 2018, and through 2020 to determine long-term effects.

**Results:**

Three years after the intervention, the GPs in our study sample prescribed 26% less opioids, 38% less benzodiazepines, and 16% less z-hypnotics. Overall prescription of PAMs decreased by 27%. The number of individuals receiving at least 90 DDD of benzodiazepines and z-hypnotics were reduced from 9 to 7 and 34 to 24 per 1000, respectively. Also, the number of individuals receiving two and three PAMs concomitantly were reduced.

**Conclusion:**

Addressing prescription practice among GPs in a community as a joint intervention, combined with addressing patients and the public may be a feasible method to obtain long-term reduction of PAM prescriptions.

**Key points**
- Non-therapeutic prescriptions of potentially addictive medications (PAMs) are both a public health concern and a frequent challenge in general practice.
- A multilevel community intervention, targeting general practitioners, patients, and the public, led to 27% reduction in prescription of PAMs.
- Both the number of daily users and concomitant use of several PAMs were reduced.
- The reduction in prescription persisted for three years.

## Introduction

Opioids, benzodiazepines and z-hypnotics (potentially addictive medications, PAMs) are widely prescribed to patients with somatic conditions, mental disorders, and addiction-related problems [1]. Used on indication, these medications are well suited to alleviate symptoms. However, prolonged use is associated with tolerance development, reduced effect, withdrawal reactions, and rebound effects upon discontinuation [1]. PAMs can also lead to increased risk for adverse health outcomes, including falls, fractures, memory impairment and vehicle accidents [2–5]. For the patients, the harms of non-therapeutic usage of PAMs are likely to outweigh any benefits obtained, and alternative non-pharmacological therapies have shown better or equivalent efficacy [6,7]. Reduced use of PAMs can therefore provide major benefits both for the individual patient and for public health.

According to the Norwegian Institute of Public Health, an especially concerning area where research efforts should be increased, concerns concomitant use of PAMs [8]. Studies have shown unfavorable tendencies in prescription of PAMs [7–9]. For opioids, prescription rates have increased across the three Nordic countries during the last decades [9]. A review of prescriptions of PAMs in the Norwegian adult population from 2005 to 2013 found that over 20% of the patients that were prescribed z-hypnotics continued to take the medications throughout a four-year period, and 10% of these patients received the medications for daily use [8]. Internationally, the increase in long-term usage of PAMs has prompted a worldwide discussion about the challenging aspects to ensure more targeted, therapeutic use.

In Norway, general practitioners (GPs) have the main responsibility for prescription, and eventually continuation and withdrawal, of PAMs [1]. A meta-synthesis of GPs experiences and perceptions of prescribing found that deliberations and decisions related to PAMs prescribing are complex and demanding [10]. GPs can differ in perceptions of their role, responsibility, and attitudes toward PAMs, and moreover, perceive a lack of alternative treatment options for these patients [10]. A crucial starting point for good treatment and therapeutic use is to ensure that GPs have good knowledge of PAMs; effect profile, indications, contraindications, side effects, and dangers of tolerance development, harmful use, and iatrogenic addiction syndrome [1]. In addition, they need good clinical communication skills and knowledge of alternative treatment methods [1]. Through careful prescriptions and, where possible, by avoiding initial prescription and instead using non-pharmacological treatment strategies, PAM prescriptions can be reduced and dependance avoided [11,12]. Both nationally and internationally, GP-targeted educational interventions, patient information letters, psychological support, and pharmacological substitutions have each been found to lead to deprescribing of PAMs [13–19]. However, follow-up in these studies have been limited to the first year or less, while the long-term effects of the interventions are unknown.

Based on an initiative from the GPs in Molde Municipality in Norway, a multilevel community intervention was initiated by the municipal chief physician in 2018, to improve the quality of prescription practice of PAMs. The aim of the intervention was to jointly increase professional and public awareness and knowledge on PAMs and their therapeutic use, and to reduce non-therapeutic prescribing. The objective of this study is to evaluate the long-term results of the multilevel community intervention, using indicators as total amount prescribed and long-term prescription.

## Material and methods

### Study setting

Molde is a municipality in the west-coast of Norway, comprising 32,000 inhabitants. In 2017, all regular GPs in this municipality were asked through surveys and interviews with the municipal chief physician to address their main concerns and goals for improvement of their practice [20]. Nearly all GPs in Molde aimed to improve their knowledge of PAMs to ensure that prescription practice was in accordance with clinical recommendations. The municipal chief physician therefore joined forces with the local GPs and designed a multilevel community intervention aiming to improve prescription practices and reduce non-therapeutic prescriptions of PAMs.

### The intervention

The multilevel community intervention was implemented as a public health intervention, not designed as a research project. The intervention was conducted in 2018 and consisted of several parts, targeting both the GPs, patients, and the public. Identical routines for prescriptions, accompanied by adapted medical note templates, were implemented among all the GP offices. Upon prescription renewal, GPs were encouraged to convert the patient usage of PAM into the average daily dosage, and to have a face-to-face consultation with the patient. Patients received information about therapeutic use of PAMs, tapering recommendations, and non-pharmacological treatment options during consultations, upon prescriptions renewal, and through patient letters. The public awareness was raised through information provided in the local newspaper and at the municipality’s website. Further details are reported in the Supplementary File (1).

### Recruitment of participating GPs to the research project

Two years after the multilevel community intervention was implemented in 2018, all 36 GPs in Molde Municipality were invited to participate in this follow-up evaluation project by contributing their anonymized prescription data on PAMs for the period 2017–2027. Of the 36 GPs, seven had changed their workplace, one retired, two did not answer, and 26 agreed to participate.

### Ethics

All participants received information about the study and on voluntary participation, in line with standards given by the Norwegian Center for Research Data (NSD). GPs who consented to participate in the study provided a written authorization to obtain their prescription data from the Norwegian Prescription Database. According to the Regional Committee for Medical and Research Ethics, this project did not require further ethical approval (Reference 230089, provided on February 05, 2021).

### Study variables

Prescription data for each of the consenting physicians were obtained for the time-period 01 January 2017–31 December 2020, covering opioids (ATC code N02A), benzodiazepines and benzodiazepine derivates (ATC codes N05BA, N05CD and N03AE), and z-hypnotics (ATC code N05CF). As several of the physicians had periods of absence from practice, prescription data for each physician were used only for months where they were working as a GP. Average amounts prescribed for each of the above-mentioned medication groups were calculated using defined daily doses (DDD) for each physician for each year [8]. These numbers were subsequently divided by the number of patient-years provided for (i.e. average size of the physicians’ patient population in the relevant year multiplied by the number of months in practice and divided by 12). We similarly calculated the average number of DDDs per patient per year prescribed excluding any palliative prescriptions to terminal patients (reimbursement code § 2–90). For each prescription, the patient’s sex and birth year was available. However, we did not have information on the distribution of age and sex among each physician’s regular patients. To be able to compare prescriptions between sexes and age groups, we therefore assumed the distribution in each patient population to be similar to the distribution in the general population in Molde. We initially categorized age in 20-year bands but chose to merge groups 0–19 and 20–39 years of age, as the number of DDDs in each of these groups were low. We calculated the number of DDDs prescribed for each patient, and for each physician, calculated the number of patients per 1000 patients per year who received 10 or less, 11–30, 31–90, or more than 90 DDDs, respectively, of each group of PAMs. We also calculated how many patients received prescriptions from only one, two or all three groups of PAMs, per 1000 patients in the physician’s patient population per year. To be able to compare the time trend in our study sample to national trends, we also retrieved publicly available national prescriptions data as well as population size in five-year age groups for the same ATC-codes as included in our study [21].

### Statistics

We performed several analyses to evaluate changes in the prescriptions over time, details are outlined in the Supplementary File. We first graphed the average unadjusted number of patients receiving categories of DDDs of PAMs per year, weighted by person-time. Second, we estimated concomitant use of different PAMs (i.e. opioids, benzodiazepines, or z-hypnotics) each year. Third, for the main results, we estimated unadjusted size of and changes in prescriptions from 2017 to 2020 using a linear mixed model with random intercept to account for dependence in observations within physicians. To communicate these results, we also calculated the magnitude of change relative to prescriptions in 2017 by simple arithmetic. Fourth, we graphed the estimated number of DDDs per patient per year within groups of age and sex for each PAM. We chose not to present CIs for these numbers, as there is substantial uncertainty about age and sex distributions among all patients. Fifth, to assess whether the difference in prescriptions over time depended on patients’ age or sex, we performed additional analyses adjusting for age group and sex and used likelihood ratio (LR) tests to compare models with or without interaction terms between time and age group or sex, respectively. Finally, we compared the prescriptions in our study sample to national trends, using Poisson regression analyses adjusted for sex and age in five-year categories. In additional analyses, we first excluded palliative prescriptions and second estimated changes using oral morphine equivalent doses of opioids. All CI are set to 95%. Data were imported to and analyzed using STATA 16 and 17.

## Results

Our study sample includes data from 20 GPs in 2017, increasing to 25 GPs in 2020, as one or more GPs were absent each year (Table 1). Each GP had on average 1147 patients (standard deviation (SD) 342) in their patient population in 2017, decreasing slightly to 1086 patients (SD 293) in 2020. Overall, assuming the patient population not to include individuals from other municipalities, our data covers between 67 and 85% of the population.

**Table 1.** Key information about what the study sample covers.

	2017	2018	2019	2020
Number of physicians present (n)	20	23	24	25
Population in Molde (n)	31,870	31,895	31,976	31,967
Total observation time (patient-years)	21,394	24,772	27,046	25,705
Percentage of population covered by study sample (%)	67.1	77.7	84.6	80.4
Average number of patients in regular patient population (mean (SD))	1147 (342)	1151 (290)	1143 (278)	1086 (293)

Around 5% of opioid prescriptions were for palliative patients, while 0.6–2.4% of benzodiazepine prescriptions and only up to 0.5% of z-hypnotic prescriptions were for palliative patients.

**Figure 1.**
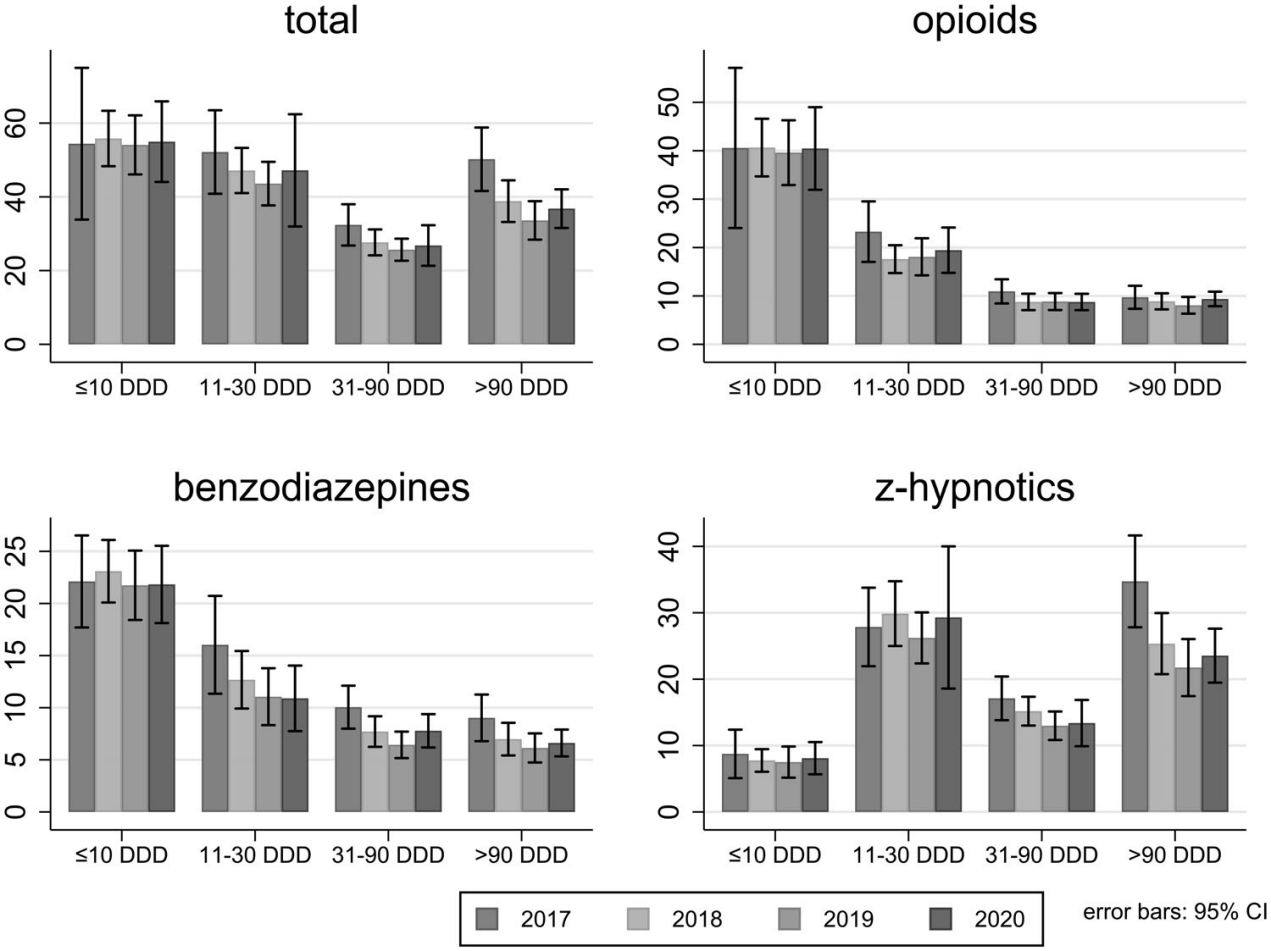
Number of patients per 1000 patients per year receiving from ≤10 to >90 DDDs of opioids, benzodiazepines, and z-hypnotics.

For opioids and benzodiazepines, GPs most often prescribed 10 DDD or less per patient per year (Figure 1). The number of patients receiving different amounts of opioids was fairly constant from 2017 to 2020. The number of patients receiving 31–90 DDDs of benzodiazepines decreased from 16 (95% CI 11–21) per 1000 patients in 2017 to 11 (95% CI 8–14) in 2020 and the number of patients receiving more than 90 DDDs decreased from 9 (95% CI 7–11) per 1000 patients in 2017 to 7 (95% CI 5–8) per 1000 patients in 2019. The number of patients receiving large amounts of z-hypnotics also decreased over time, with 34 patients per 1000 (95% CI 28–42) receiving more than 90 DDDs in 2017 compared to 24 (95% CI 19–28) per 1000 in 2020.

The number of patients receiving all three groups of PAMs was low and stable (5 per 1000 in 2017, 4 per 1000 in 2018 to 2020, Supplementary Table S1). The number of patients receiving two different groups of PAMs declined slightly from 28 per 1000 in 2017 to 26 per 1000 in 2018 and further to 24 per 1000 in 2019 and 2020.

Estimated average total prescription of PAMs was reduced with 4.5 DDD (95% CI 3.3–5.6) per patient in 2020 compared to 2017 (Table 2). This corresponds to an estimated 27% reduction. Similarly, prescriptions of opioids were reduced by 0.7 DDD (95% CI 0.1–1.2), benzodiazepines by 0.8 DDD (95% CI 0.5–1.2) and z-hypnotics by 2.9 DDD (95% CI 2.2–3.7) per patient comparing 2020 to 2017. This represents 17%, 27% and 30% estimated reduction in opioids, benzodiazepines, and z-hypnotics, respectively. There were small differences between the years 2018 to 2020 for each group of PAMs, with no clear trend over these years. Prescriptions were still slightly lower in 2019 than 2020. Results were similar when excluding palliative prescriptions (Supplementary Table S2). Opioid prescriptions were lower throughout the observation period when using oral morphine equivalent doses, while the pattern of changes over time were similar to main results (Supplementary Tables S3 and S4).

**Table 2.** Estimated average number of DDDs per patient per year and estimated changes between 2017 and years 2018 to 2020 for potentially addictive medications (PAMs) in Molde.

	Study sample in Molde				Norway
Year	DDD per patient	Change	95% CI		DDD per person
All PAMs					
2017	16.7	Reference			22.0
2018	12.3	−4.4	−5.6	−3.3	21.3
2019	11.7	−5.0	−6.2	−3.9	20.9
2020	12.3	−4.5	−5.6	−3.3	20.8
Opioids					
2017	4.1	Reference			6.1
2018	3.2	−0.9	−1.4	−0.3	6.0
2019	3.3	−0.9	−1.4	−0.3	6.0
2020	3.5	−0.7	−1.2	−0.1	5.9
Benzodiazepines					
2017	3.0	Reference			4.9
2018	2.2	−0.8	−1.1	−0.5	4.6
2019	2.1	−1.0	−1.3	−0.7	4.5
2020	2.2	−0.8	−1.2	−0.5	4.3
Z-hypnotics					
2017	9.6	Reference			11.0
2018	6.8	−2.7	−3.5	−2.0	10.7
2019	6.4	−3.2	−3.9	−2.5	10.5
2020	6.6	−2.9	−3.7	−2.2	10.6

*Notes:* For comparison, we also report DDDs prescribed per person in all of Norway. Results for Molde are based on a linear mixed model, while results for Norway are calculated using publicly available data on prescriptions (www.reseptregisteret.no) and population size (www.ssb.no/folketall) without any adjustments.

**Figure 2.**
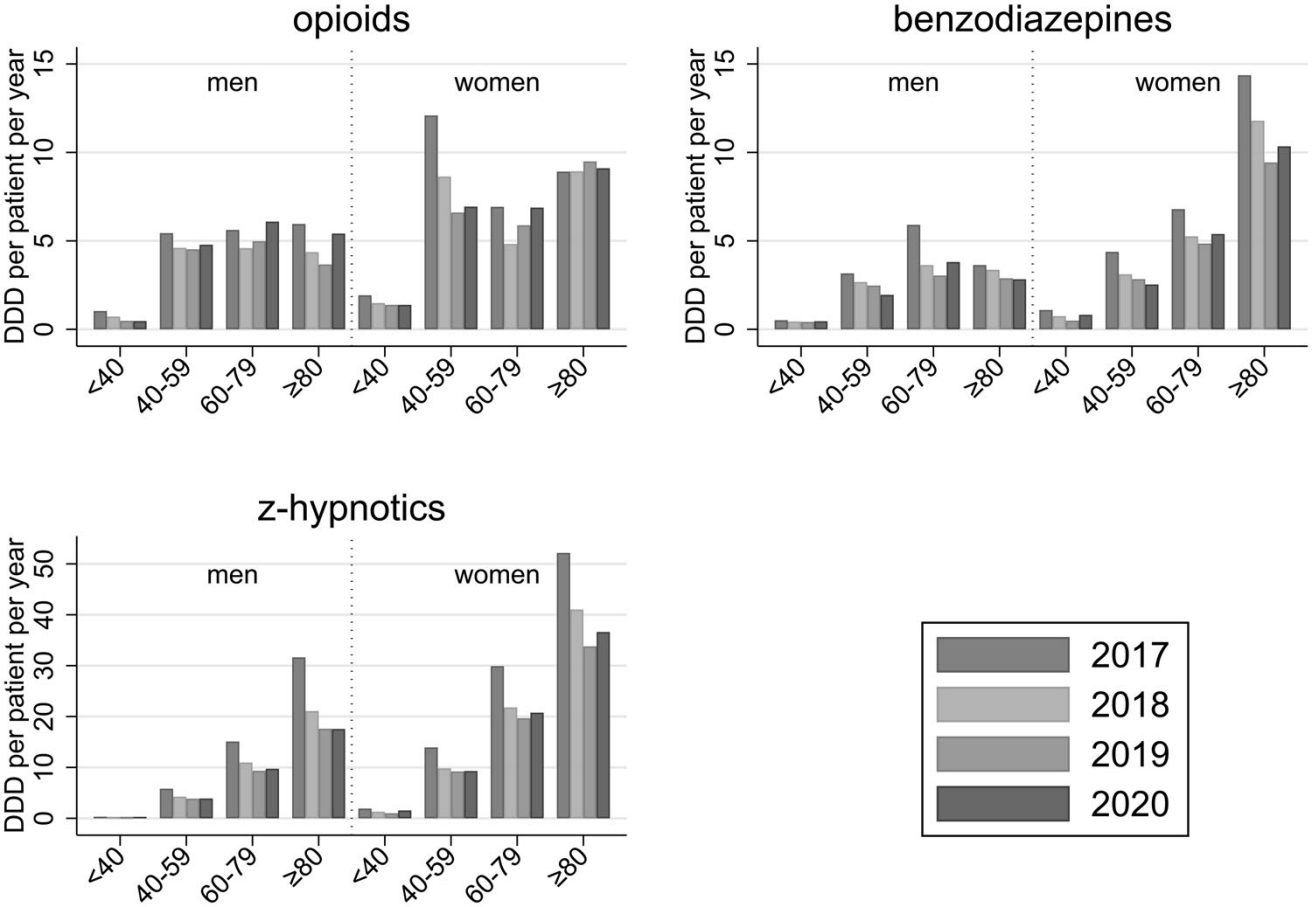
Average number of DDDs prescribed per patient per year according to sex and age group.

GPs prescribed more of each PAM to female patients compared to male patients (Figure 2). Prescriptions of benzodiazepines were notably higher among women aged 80 years and older, and prescriptions of z-hypnotics increased with age among both men and women. We found weak statistical evidence that the decline in prescriptions of z-hypnotics depended on age (LR-test *p* = .085) with a greater decline in older age (Supplementary Table S5). The statistical evidence for differences in prescription changes between age groups was weak for opioids and benzodiazepines (LR-test *p* = .9), as was the evidence for differences in changes between men and women (LR-test *p* .4–.8). We still note an observed 28% decline in prescriptions of benzodiazepines from 14.4 DDD in 2017 to 10.3 DDD in 2020 per 1000 women over 80 years of age (Figure 2).

Compared to all of Norway, the GPs in our study sample prescribed 26% less opioids (95% CI 26–27%), 38% less benzodiazepines (95% CI 37–38%) and 16% less z-hypnotics (95% CI 15–16%) in 2017 (Supplementary Table S6). For each PAM, there was a national trend of lower prescriptions for each year, most prominently so for benzodiazepines, where prescriptions were 13% lower in 2020 compared to 2017. Still, the reduction in prescriptions from 2017 to 2020 was around 20 to 30% greater in our study sample compared to all of Norway.

## Discussion

This study showed a 27% reduction in prescription of PAMs after a multilevel community intervention targeting both GPs, patients, and the general public. The reduction in prescriptions was substantial for each class of PAMs, though somewhat greater for z-hypnotics and benzodiazepines, compared to opioids. These changes clearly exceeded a national trend of lower prescription rates. While the largest decline appeared the first year after the intervention, the change sustained for the three observed years. A minor (and statistically non-significant) increase in prescriptions observed in the third year compared to the second year may represent a random fluctuation or an effect of changed prescription routines due to the Covid-19 pandemic (see below). Generally, reduced prescriptions were found across age groups and sexes. However, the prescription of z-hypnotics was substantially greater in the older age groups, where the decline also seemed stronger. For benzodiazepines, we also noted a substantially higher use among the oldest women with a pronounced decline after the intervention.

The use of registry data assured completeness of prescriptions from the participating GPs, and multilevel analyses allowed us to account for differences between the physicians and their patient populations. We still note some limitations. The study was small and only include prescriptions from the regular GPs. We cannot exclude the possibility that patients sought prescriptions from out-of-hours care or specialist health services, which would cause an overestimation of the effect. However, this is unlikely in the Norwegian healthcare setting (see Supplementary Information). Another limitation is that we do not have the age and sex distribution among all patients served by the GPs in the study, so the sex- and age-specific analyses should be interpreted with caution. Although we were able to consider which of the GPs were present at any time, extrapolation of prescriptions made when present may not be correct. However, this potential measurement error would be independent of the intervention and thus unlikely to explain the results. Of the physicians originally participating in the intervention, eight are missing for reasons presumably unrelated to the effects of the intervention. Still, there is a theoretical chance that non-participation might have led to overestimation of the effect. However, as only two out of 28 eligible GPs did not participate, such effect is expected to be small.

Non-therapeutic use of PAMs among older patients is of particular concern due to age-related pharmacokinetic and pharmacodynamic changes, multimorbidity, and polypharmacy [1,13]. The high prescriptions of benzodiazepines among women and z-hypnotics among both sexes that we found from age 80, is thus of concern. Our intervention decreased the prescription of both these medications, and for z-hypnotics, the decline seemed strongest among the oldest patients. Our findings correspond with previous research suggesting that mixed interventions could yield discontinuation rates of benzodiazepines and other hypnotics between 27% and 80% among older people [13]. However, since the sustainability of these interventions was previously indetermined [13], and since GPs have experienced elderly patients rejecting proposed medication changes as a particular challenge [22], our finding that the intervention had long-term effects on prescription rates for older patients is important.

GPs find the process of prescribing PAMs complex and demanding [10,19] and have called for more knowledge, tools for a practical approach, and a clear overview of effective reduction strategies [19,23,24]. This was also the starting point for our intervention. At the same time, GPs can perceive deprescribing interventions initiated by the regional authorities as a type of control or cost reduction tool interfering with their clinical autonomy [22]. The fact that the initiative came from the GPs themselves, leading to a collective, multilevel approach concomitantly targeting GPs, patients, and the public is likely to have increased their motivation and dedication. In our intervention, the municipality chief physician offered the GPs support, strategies, and tools that were based on a thorough understanding of how prescribing decisions are made. The patients received information through several channels and a face-to-face consultation with their GP, which allowed for genuine user involvement [1]. In line with recommendations [25], a detailed description of our intervention is included as a supplement to this paper to allow replication.

During the Covid-19 pandemic, communication between the GPs and the patients, including renewal of PAM prescriptions, were quite abruptly digitalized to a high degree. This mean that the intervention component of providing face-to-face consultations was not carried out in 2020, potentially affecting the GPs’ prescribing practice. Prescribing without a patient consultation has been found to be one of the main predictors of high-volume prescribing of PAMs [26]. At the individual GP level, competing priorities and time pressure have been reported as barriers for putting medication changes on the agenda [22]. Although this multilevel community intervention reduced the GPs’ prescriptions of PAMs, we believe that the intervention can be even more successful in the long run with periodic public reminders and training for GPs as this could maintain awareness. In a scale-up of the intervention, we recommend that provider training in non-technical therapeutic skills is implemented, as it may enhance the efficacy of prescriber education programs [27].

## Supplementary Material

Supplemental Material

Supplemental Material
