# Supplementary material for: Prescription of potentially addictive medications after a multilevel community intervention in general practice
Source: Scand J Prim Health Care. 2023 Jan 20;41(1):61–8. doi: 10.1080/02813432.2023.2168125 (PMC10088976; doi:10.1080/02813432.2023.2168125)
Supplement: Supplemental Material [file IPRI_A_2168125_SM9983.docx]

**
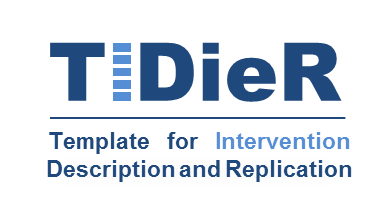
The TIDieR (Template for Intervention Description and Replication) Checklist*:**

Information to include when describing an intervention and the location of the information

|  |  |  |
| --- | --- | --- |

**BRIEF NAME**

A multilevel coomunity intervention on prescribing of potentially addictive medications (PAMs) in general practice.

**WHY**

In 2017, all regular general practitioners (GPs) in Molde municipality were asked through surveys and interviews with the municipal chief physician to address their main concerns and goals for improvement of their practice. Based on this initiative from the GPs, a multilevel educational intervention was initiated by the municipal chief physician in 2018, to improve their knowledge of PAMs to ensure that prescription practice was in accordance with clinical recommendations. The multilevel educational intervention was implemented as a public health intervention and not designed as a research project.

**WHAT AND WHO**

The intervention consisted of several parts, targeting the GPs, patients, and the public.

*Components targeting the GPs*

- Feedback: Before implementing the intervention, the municipal chief physician prepared summaries of the prescription practice for each GP and the six GP offices in Molde. These summaries were reviewed with the GPs in plenary at each office, so that everyone received their baseline status at start-up. Corresponding results were also presented after 6, 12, and 24 months.
- Increased awareness: The municipal chief physician increased the GPs awareness of non-medical treatment options, tapering, and if necessary, the possibility of using short-acting instead of long-acting benzodiazepine and z-hypnotics.
- [Template for tapering PAMs](https://helserespons.no/web/doku/1521832829_693.docx): The GPs were involved in the development of a tapering template, that was identical for the six GP offices.
- Journal template: A structured medical checklist for PAMs was developed, which the GPs could use during patient consultation. This checklist contained questions such as: does the patient meet the recommendation for tapering of PAM?
- Average daily dose: When a patient contacted the GP office upon renewal of their PAM prescription, the offices were encouraged to convert to the average daily dosage of PAMs. This conversion could be conducted by the GPs or by the secretaires.
- Face-to-face consultations: When a patient contacted the GP office upon renewal of their PAM prescription, they were encouraged to make an appointment with their GP.
- Identical routines: The components described above were implemented among all the GP offices to provide collective approach.
- Support: The municipal chief physician offered support to the GPs by request.

*Components targeting the patients*

- Face-to-face consultation: Patients received information (about therapeutic use of PAMs, tapering recommendations, and non-pharmacological treatment options) from their GP during consultations.
- Patient letters: The municipality chief physician developed in collaboration with the GPs, patient information letters about therapeutic use of PAMs, tapering recommendations, and non-pharmacological treatment options:
- [Patient letter](https://helserespons.no/web/doku/1519074462_690.docx): For benzo and z-hypnotics.
- [Patient letter](https://helserespons.no/web/doku/1519074428_689.docx): For opioids.
- The municipal chief physician developed courses about PAMs for other health care workers in the municipal health service, such as home nursing.

*Components targeting the public*

- [A reportage in the local newspaper](https://www.rbnett.no/nyheter/i/jzpo00/slik-vil-de-hjelpe-pasienter-uten-at-de-blir-medisinavhengige): The GPs and the municipality chief physician were interviewed in the local newspaper about PAMs. The main message was to increase awareness of PAMs and the public health intervention. One of the key messages, was that the patients should not feel ashamed of using PAMs. The point was to use it correctly - the right medicine, in the right dose, at the right time.
- [Municipality’s website](https://www.molde.kommune.no/helse-og-omsorg/psykisk-helse-og-rus/rask-psykisk-helsehjelp/rask-psykisk-helsehjelp/): Information was provided through the municipality´s website and informed the public about low-threshold treatment alternative available in the municipality, such as non-medical treatments and cognitive therapy for sleep disorder, anxiety and depression.
- Non-pharmacological c[ourses](https://www.molde.kommune.no/helse-og-omsorg/psykisk-helse-og-rus/rask-psykisk-helsehjelp/rask-psykisk-helsehjelp/): The municipality offered courses to manage anxiety and sleep disturbances, and these courses were available for the public, free of charge.

**WHERE**

The general practices in Norway are mainly independent, physician-owned small businesses that provide primary medical care under a collective agreement with the municipality and are mainly funded by HELFO (The Norwegian Health Economics Administration). The services are tax-financed and administered by the HELFO, including reimbursement of expenses for drugs eligible for subsidy. In the Norwegian healthcare setting, the out-of-hours services are largely employed by GPs working within the same municipality

**MODIFICATIONS**

No adjustments were made to individual GPs or selected patient groups. The intervention was planned to be universal, regardless of patient characteristics.

**HOW WELL**

During the Covid-19 pandemic, PAM prescriptions and communication between the GPs and the patients were digitalized on a larger scale. This mean that the intervention component of providing face-to-face consultations were not carried out in 2020, which might have affected the GPs’ prescribing practice.

**FROM PUBLIC HEALTH INTERVENTION TO RESEARCH**

This multilevel community intervention was implemented as a public health intervention, not designed as a research project. To allow replication of the analyses performed, we provide additional information on the statistical analyses conducted.

We performed several analyses to evaluate changes in the prescriptions over time. We first graphed the average number of patients receiving from up to ten defined daily doses (DDDs) to more than 90 DDDs of each PAM and summarized for all PAMs per 1000 patients per year. We calculated confidence intervals (CIs) using the t-distribution with one observation per physician per year, without any adjustment, weighted by the number of person-years each physician contributed. In this analysis, we included the time in which each physician was present, without regard to differences between those present and those not present in different years. Second, we similarly estimated the number of patients receiving two or three different groups of PAMs (i.e. opioids, benzodiazepines or z-hypnotics) each year. Third, for the main results, we compared the number of DDDs per patient per year between baseline (2017) and each of the three years following the intervention (2018-2020). This was done for each group of PAMs separately as well as for all PAMs summarized. We used a linear mixed model with random intercept to account for dependence in observations within physicians. We did not adjust for any further variables. The regression equation is thus (j denotes the physician and ij denotes the different years):


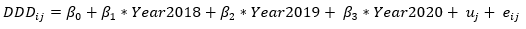


To assess whether there was a trend from 2018 to 2020, indicating an attenuation of the effect of the intervention with time, we additionally performed similar models excluding the year 2017. In these analyses, years were treated as a continuous variable, meaning we estimated the linear trend over the two subsequent years after the intervention.

We subsequently used postestimation command margins to estimate the average DDDs prescribed for each year. When assessing the assumptions of linear regression, we found some deviation for normality for opioids, and heteroskedasticity for each group of PAMs. Also, the prescription levels for each physician seem to have a somewhat skewed distribution. Fourth, we graphed the estimated number of DDDs per patient per year within groups of age and sex for each PAM. We chose not to present CIs for these numbers, as there is likely substantial error in the estimated age and sex distribution for each physician, while the distribution in the total observed patient population is likely to be close to the distribution in Molde municipality. Fifth, to assess whether the difference in prescriptions over time depended on patients’ age or sex, we performed additional analyses adjusting for age group and sex and used LR-tests to compare models with or without interaction terms between time and age group or sex, respectively. Age group was included as indicator variables in these analyses. Finally, we summarized prescriptions from all included GPs and compared the prescription trends in our study sample to those in Norway, using Poisson regression analyses adjusted for sex and age in 5-year categories. Again, we assumed the age and sex distribution in our study sample to be the same as that in Molde municipality. Main results include all prescriptions, but we performed additional analyses excluding palliative prescriptions. All CI are set to 95%. Data were imported to and analyzed using STATA 16 and 17.
