# Supplementary material for: Prescription of potentially addictive medications after a multilevel community intervention in general practice
Source: Scand J Prim Health Care. 2023 Jan 20;41(1):61–8. doi: 10.1080/02813432.2023.2168125 (PMC10088976; doi:10.1080/02813432.2023.2168125)
Supplement: Supplemental Material [file IPRI_A_2168125_SM9982.docx]

**Supplementary table S1: Concomitant use of potentially addictive medications (PAMs).**

Estimated number of patients per 1000 per year who received one, two or three classes of PAMs, respectively. The three classes of PAMs included in the analyses are opioids, benzodiazepines or benzodiazepine derivates, and z-hypnotics.

| **Year** |  | **Patients per 1000 per year** | **95% CI** | | |
| --- | --- | --- | --- | --- | --- |
| 2017 | One class of PAMs | 142 | 123 | - | 161 |
|  | Two classes of PAMs | 28 | 24 | - | 33 |
|  | Three classes of PAMs | 5 | 3 | - | 7 |
| 2018 | One class of PAMs | 135 | 120 | - | 150 |
|  | Two classes of PAMs | 26 | 22 | - | 30 |
|  | Three classes of PAMs | 4 | 3 | - | 6 |
| 2019 | One class of PAMs | 129 | 116 | - | 142 |
|  | Two classes of PAMs | 24 | 20 | - | 28 |
|  | Three classes of PAMs | 4 | 2 | - | 5 |
| 2020 | One class of PAMs | 128 | 110 | - | 146 |
|  | Two classes of PAMs | 24 | 20 | - | 28 |
|  | Three classes of PAMs | 4 | 3 | - | 5 |

**Supplementary table S2: Estimated number of defined daily doses (DDDs) of all potentially addictive medications (PAMs).**

Opioids, benzodiazepines and benzodiazepine derivates, and z-hypnotics prescribed per year and estimated change in prescription from 2017. Results from a linear mixed model with a random intercept, with years nested within physicians. Prescriptions with refund code §2 -90 have been excluded.

|  | **Year** | **DDD per patient** | **Change** | **95% CI** | | | ***p*-value** | ***p* for trend 2018-2020** |
| --- | --- | --- | --- | --- | --- | --- | --- | --- |
| All PAMs | 2017 | 16.5 | 0.0 | Reference | | |  |  |
|  | 2018 | 12.1 | -4.4 | -5.6 | - | -3.2 | <0.001 | 0.735 |
|  | 2019 | 11.5 | -5.0 | -6.2 | - | -3.8 | <0.001 |  |
|  | 2020 | 12.0 | -4.5 | -5.6 | - | -3.3 | <0.001 |  |
| Opioids | 2017 | 3.9 | 0.0 | Reference | | |  |  |
|  | 2018 | 3.1 | -0.8 | -1.4 | - | -0.3 | 0.003 | 0.104 |
|  | 2019 | 3.1 | -0.8 | -1.3 | - | -0.3 | 0.003 |  |
|  | 2020 | 3.3 | -0.6 | -1.1 | - | -0.1 | 0.031 |  |
| Benzodiazepines | 2017 | 3.0 | 0.0 | Reference | | |  |  |
|  | 2018 | 2.2 | -0.8 | -1.1 | - | -0.5 | <0.001 | 0.381 |
|  | 2019 | 2.0 | -1.0 | -1.3 | - | -0.7 | <0.001 |  |
|  | 2020 | 2.1 | -0.9 | -1.2 | - | -0.6 | <0.001 |  |
| Z-hypnotics | 2017 | 9.6 | 0.0 | Reference | | |  |  |
|  | 2018 | 6.8 | -2.8 | -3.5 | - | -2.0 | <0.001 | 0.254 |
|  | 2019 | 6.4 | -3.2 | -3.9 | - | -2.5 | <0.001 |  |
|  | 2020 | 6.6 | -3.0 | -3.7 | - | -2.2 | <0.001 |  |

**Supplementary table S3: Defined daily doses (DDDs) of opioids included in the study.**

Oral morphine equivalent doses used in calculations, and the factor with which each opioid prescription was multiplied to calculate the DDDs of oral morphine equivalents.

| **ATC code** | **Name** | **Combined with** | **Administ-ration** | **DDD** | | | **Oral morphine equivalents** | **Factor** |
| --- | --- | --- | --- | --- | --- | --- | --- | --- |
| N02AA01 | [Morphine](https://www.whocc.no/atc_ddd_index/?code=N02AA01&showdescription=yes) |  | oral | 100 | mg | 100 | | 1 |
| N02AA03 | Hydromorhpone |  | oral | 20 | mg | 80 | | 0.8 |
| N02AA05 | [Oxycodone](https://www.whocc.no/atc_ddd_index/?code=N02AA05&showdescription=yes) |  | oral | 75 | mg | 112.5 | | 1.125 |
| N02AA55 | [Oxycodone](https://www.whocc.no/atc_ddd_index/?code=N02AA05&showdescription=yes) | Naloxone | oral | 75 | mg | 112.5 | | 1.125 |
| N02AB01 | Ketobemidone |  | oral | 50 | mg | 50 | | 0.5 |
| N02AB02 | Pethidine |  | rectal | 400 | mg | 40 | | 0.4 |
| N02AB03 | Fentanyl |  | trans-dermal | 17 | µg/h | 40.8 | | 0.408 |
| N02AD01 | Pentazocine |  | oral | 200 | mg | 44 | | 0.44 |
| N02AE01 | Buprenorphine |  | trans-dermal | 7 | µg/h | 15.54 | | 0.1554 |
| N02AG02 | Ketobemidone | Spasmolytics | rectal | 50 | mg | 50 | | 0.5 |
| N02AJ06 | [Codeine](https://www.whocc.no/atc_ddd_index/?code=R05DA04) | Paracetamol | oral/ rectal | 90-120 | mg | 13.5-18 | | 0.135-0.18* |
| N02AJ13 | [Tramadol](https://www.whocc.no/atc_ddd_index/?code=N02AX02&showdescription=yes) | Paracetamol | oral | 150 | mg | 15 | | 0.15 |
| N02AX02 | [Tramadol](https://www.whocc.no/atc_ddd_index/?code=N02AX02&showdescription=yes) |  | oral | 300 | mg | 30 | | 0.3 |
| N02AX06 | Tapentadol |  | oral | 400 | mg | 160 | | 1.6 |

* In the Norwegian Prescription Registry, mg codeine per DDD differs according to how much paracetamol it is combined with. One DDD of sales names Altermol, Pinex Forte and Pinex Major contain 90 mg codeine and 1500 mg paracetamol, while one DDD of sales name Paralgin Forte contains 120 mg codeine and 1600 mg paracetamol.

**Supplementary table S4: Estimated number of defined daily doses (DDDs) of opioids prescribed per year and estimated change in prescription from 2017.**

Opioid prescriptions have been recalculated using oral morphine equivalent doses. Results from a linear mixed model with a random intercept, with years nested within physicians.

|  | **Year** | **DDD per patient** | **Change** | **95% CI** | | | ***p*-value** | ***p* for trend 2018-2020** |
| --- | --- | --- | --- | --- | --- | --- | --- | --- |
| Opioids | 2017 | 1.9 | -0.4 | Reference | | |  |  |
|  | 2018 | 1.5 | -0.4 | -0.8 | - | 0.0 | 0.058 | 0.517 |
|  | 2019 | 1.4 | -0.5 | -0.9 | - | -0.0 | 0.028 |  |
|  | 2020 | 1.5 | -0.3 | -0.7 | - | 0.1 | 0.109 |  |

**Supplementary table S5: Estimated change in number of defined daily doses (DDDs) prescribed per 1000 patients per year of z-hypnotics compared to 2017.**

Results from a linear mixed model with a random intercept and years nested within physicians, analyzed separately by age groups, adjusted for sex.

| **Age groups** | **Year** | **DDD** | **Change** | **95% CI** | | | ***p*-value** |
| --- | --- | --- | --- | --- | --- | --- | --- |
| <40 years | 2017 | 1.1 | 0.0 | Reference | | |  |
|  | 2018 | 0.8 | -0.4 | -1.0 | - | 0.3 | 0.292 |
|  | 2019 | 0.6 | -0.5 | -1.2 | - | 0.2 | 0.143 |
|  | 2020 | 0.9 | -0.2 | -0.9 | - | 0.5 | 0.554 |
| 40-59 years | 2017 | 9.7 | 0.0 | Reference | | |  |
|  | 2018 | 6.8 | -2.9 | -5.0 | - | -0.9 | 0.005 |
|  | 2019 | 6.4 | -3.3 | -5.4 | - | -1.2 | 0.002 |
|  | 2020 | 6.3 | -3.5 | -5.5 | - | -1.4 | 0.001 |
| 60-79 years | 2017 | 22.1 | 0.0 | Reference | | |  |
|  | 2018 | 15.1 | -7.0 | -10.2 | - | -3.9 | <0.001 |
|  | 2019 | 14.0 | -8.1 | -11.2 | - | -4.9 | <0.001 |
|  | 2020 | 14.6 | -7.5 | -10.6 | - | -4.4 | <0.001 |
| 80+ years | 2017 | 38.7 | 0.0 | Reference | | |  |
|  | 2018 | 29.0 | -9.7 | -17.3 | - | -2.0 | 0.013 |
|  | 2019 | 25.9 | -12.8 | -20.5 | - | -5.2 | 0.001 |
|  | 2020 | 27.2 | -11.5 | -19.2 | - | -3.9 | 0.003 |

**Supplementary table S6: Associations of year and sample with defined daily doses (DDDs) of potentially addictive medications (PAMs).**

Estimated associations of year, sample (study sample in Molde compared to all of Norway) and their interaction terms with number of defined daily doses (DDD) prescribed for all potentially addictive medications (PAMs), opioids, benzodiazepines and benzodiazepine derivates, and z-hypnotics, respectively. Results from Poisson regression models adjusted for age in 5-year bands and sex. For each type of medication, prescriptions in Norway in 2017 are used as reference values. IRR (incidence rate ratio) for years compare 2017 to later years in Norway, IRR for Molde compare Molde in 2017 to Norway in 2017, while interaction terms compare changes over time in Molde to changes over time in Norway.

| **Medication** | **Exposure variable** | | **IRR** | **95% CI** | | |
| --- | --- | --- | --- | --- | --- | --- |
| All PAMs | Year | 2017 | 1.00 | Reference | | |
|  |  | 2018 | 0.96 | 0.96 | - | 0.96 |
|  |  | 2019 | 0.93 | 0.93 | - | 0.93 |
|  |  | 2020 | 0.92 | 0.92 | - | 0.92 |
|  | Sample | Norway | 1.00 | Reference | | |
|  |  | Study sample | 0.77 | 0.76 | - | 0.77 |
|  | Interaction term | 2018#study sample | 0.77 | 0.76 | - | 0.78 |
|  |  | 2019#study sample | 0.71 | 0.70 | - | 0.71 |
|  |  | 2020#study sample | 0.77 | 0.76 | - | 0.77 |
| Opioids | Year | 2017 | 1.00 | Reference | | |
|  |  | 2018 | 0.97 | 0.97 | - | 0.97 |
|  |  | 2019 | 0.96 | 0.96 | - | 0.96 |
|  |  | 2020 | 0.94 | 0.94 | - | 0.94 |
|  | Sample | Norway | 1.00 | Reference | | |
|  |  | Study sample | 0.74 | 0.73 | - | 0.74 |
|  | Interaction term | 2018#study sample | 0.79 | 0.78 | - | 0.79 |
|  |  | 2019#study sample | 0.75 | 0.74 | - | 0.75 |
|  |  | 2020#study sample | 0.83 | 0.83 | - | 0.84 |
| Benzodiazepines | Year | 2017 | 1.00 | Reference | | |
|  |  | 2018 | 0.94 | 0.94 | - | 0.94 |
|  |  | 2019 | 0.90 | 0.90 | - | 0.90 |
|  |  | 2020 | 0.87 | 0.87 | - | 0.87 |
|  | Sample | Norway | 1.00 | Reference | | |
|  |  | Study sample | 0.62 | 0.62 | - | 0.63 |
|  | Interaction term | 2018#study sample | 0.79 | 0.78 | - | 0.80 |
|  |  | 2019#study sample | 0.72 | 0.71 | - | 0.73 |
|  |  | 2020#study sample | 0.80 | 0.79 | - | 0.81 |
| Z-hypnotics | Year | 2017 | 1.00 | Reference | | |
|  |  | 2018 | 0.96 | 0.96 | - | 0.96 |
|  |  | 2019 | 0.93 | 0.93 | - | 0.93 |
|  |  | 2020 | 0.93 | 0.93 | - | 0.93 |
|  | Sample | Norway | 1.00 | Reference | | |
|  |  | Study sample | 0.84 | 0.84 | - | 0.85 |
|  | Interaction term | 2018#study sample | 0.76 | 0.75 | - | 0.76 |
|  |  | 2019#study sample | 0.68 | 0.67 | - | 0.68 |
|  |  | 2020#study sample | 0.72 | 0.72 | - | 0.73 |
